# Local Structure Awareness-Based Retinal Microaneurysm Detection with Multi-Feature Combination

**DOI:** 10.3390/biomedicines10010124

**Published:** 2022-01-07

**Authors:** Jiakun Deng, Puying Tang, Xuegong Zhao, Tian Pu, Chao Qu, Zhenming Peng

**Affiliations:** 1 School of Information and Communication Engineering, University of Electronic Science and Technology of China, Chengdu 611731, China; 201921050407@std.uestc.edu.cn (J.D.); xgzhao@uestc.edu.cn (X.Z.); putian@uestc.edu.cn (T.P.); 2 School of Optoelectronic Science and Engineering, University of Electronic Science and Technology of China, Chengdu 611731, China; 3 Laboratory of Imaging Detection and Intelligent Perception, University of Electronic Science and Technology of China, Chengdu 611731, China; 4 Department of Ophthalmology, Sichuan Academy of Medical Sciences and Sichuan Provincial People’s Hospital, Chengdu 610072, China; lucyjeffersonqu@hotmail.com

**Keywords:** diabetic retinopathy, microaneurysm detection, feature extraction, fundus image analysis

## Abstract

Retinal microaneurysm (MA) is the initial symptom of diabetic retinopathy (DR). The automatic detection of MA is helpful to assist doctors in diagnosis and treatment. Previous algorithms focused on the features of the target itself; however, the local structural features of the target and background are also worth exploring. To achieve MA detection, an efficient local structure awareness-based retinal MA detection with the multi-feature combination (LSAMFC) is proposed in this paper. We propose a novel local structure feature called a ring gradient descriptor (RGD) to describe the structural differences between an object and its surrounding area. Then, a combination of RGD with the salience and texture features is used by a Gradient Boosting Decision Tree (GBDT) for candidate classification. We evaluate our algorithm on two public datasets, i.e., the e-ophtha MA dataset and retinopathy online challenge (ROC) dataset. The experimental results show that the performance of the trained model significantly improved after combining traditional features with RGD, and the area under the receiver operating characteristic curve (AUC) values in the test results of the datasets e-ophtha MA and ROC increased from 0.9615 to 0.9751 and from 0.9066 to 0.9409, respectively.

## 1. Introduction

The number of diabetes patients worldwide is gradually increasing and, with the progression of diabetes, patients may develop DR, which may eventually cause vision loss or even blindness [1]. MA is the initial symptom of DR, and the early identification and timely treatment of retinal MA can prevent further progression of DR. Therefore, it is of great medical significance to realize the automatic detection of MA and assist doctors in the diagnosis of retinal lesions through computer technology.

**Figure 1:**
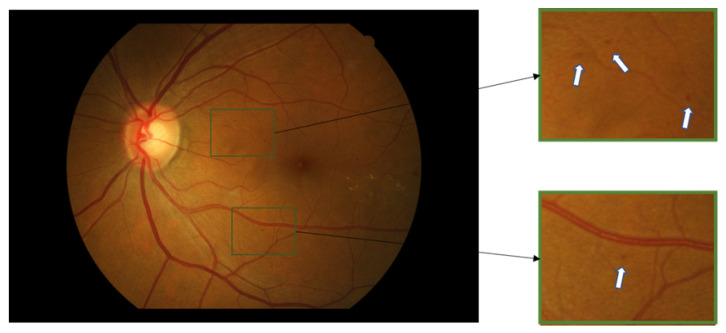
An example of a color fundus image with MAs. The areas with MAs are identified with a green box and zoomed in on the right side of the image, the dark blobs that the white arrow points to are MAs.

Color fundus images are the primary way ophthalmologists assess retinal lesions, they judge whether the retina is normal and the grade of DR by visually observing whether there are microaneurysms, hard exudations, soft exudations, hemorrhages, and neovessels in the color fundus images [2]. MA occupies only a few pixels in fundus images and has low local contrast, as shown in Figure 1. At the same time, due to factors, such as the environment and equipment, color fundus images often have different brightness, contrast, and color. Artificial detection of MA is time-consuming, with low accuracy, and easily leads to ophthalmologist fatigue. Therefore, many researchers have studied the automatic detection of MA.

The current mainstream MA detection algorithm [3,4,5,6,7] can be briefly summarized as the following three steps: preprocessing, candidate extraction, and candidate classification. The preprocessing methods mainly include color correction, contrast enhancement, reflective elimination, and other image enhancement operations. The main purpose of pretreatment is to better observe the lesions in the retina and prepare for subsequent algorithms. Candidate extraction is primarily to extract image blocks that may contain MA from color fundus images, mainly using morphology, filtering, and other methods. In the step of candidate classification, the accurate detection of MA can be realized by extracting hand-crafted features of each candidate and classifying them with a machine learning classifier.

In this work, we first performed color correction on fundus images, and then used morphological methods to extract MA candidates. We found that MAs and blood vessels were mainly present in the candidate region, and traditional features based on the candidate area are not sufficiently capable and interpretable of distinguishing between them. To make up for the lack of traditional features, a novel local structure feature called ring gradient descriptor (RGD) is proposed, which scans the background around the target in an annular way to find the region most similar to the target and calculates the similarity between the region and the target. Then, a combination of RGD and the salience and texture features of candidate objects is used by Gradient Boosting Decision Tree (GBDT) for the final candidate classification.

The major contributions of this paper can be summarized as follows.

1. A novel method is proposed for the accurate and reliable detection of microaneurysms with the possibility of applying this method in large screening setups.
2. A simple candidate extraction algorithm based on morphology is proposed to extract the potential MA in fundus images.
3. A new local structure feature RGD is proposed that can describe the local structure of object and its surrounding background and improve the classification performance.

## 2. Related Works

The detection algorithm of MA can be divided into the physical model-based method, classifier-based method, and deep learning-based detection methods.

Physical model-based MA detection methods are mainly based on the physical characteristics of retinal MA. Joshi et al. [8] employed morphological methods to enhance fundus images and remove blood vessels and then extracted MAs. Zhang et al. [9] proposed a feature-transfer network and local background suppression for MA detection by using the similarity matrix of feature distances to measure the difference between background noise and retinal objects to suppress the local background. Quellec et al. [10] did not perform MA detection in color fundus images but used a lesion template for MA matching in its wavelet-transformed images.

The classifier-based method is the most prevalent method at present, the main process of this method includes candidate extraction, feature extraction, and candidate classification. Orlando et al. [3] employed morphological reconstruction to extract MA candidates. In the process of feature extraction, they constructed a Convolutional Neural Network (CNN) model to extract depth features and combined it with color features, textural features, and geometrical features for candidates classification. Dashtbozorg et al. [4] used a gradient-weighting technique and an iterative thresholding approach to extract MA candidates and used the response of local convergence index filters and the salience of the candidate area for classification.

Melo et al. [5] used a sliding band filter for MA enhancement and they also used the filter response and the salience of the candidate area for classification. Antal and Hajdu [6] proposed an ensemble-based framework for MA detection; they selected the optimal results under different preprocessing and candidate extraction methods. Shah et al. [7] removed blood vessels from the green channel and extracted MA candidates using a local thresholding technique. They classified MAs and non-MAs based on statistical features.

The deep learning-based detection method mainly regards MA detection as a segmentation task. Xu et al. [11] improved the U-Net model and achieved pixel-level segmentation of MA. Liao et al. [12] proposed a novel deep convolutional encoder–decoder network for MA detection. Budak et al. [13] used a CNN trained with preprocessed RGB patches to classify MA patches and non-MA patches.

Due to the complex structure of retina and the uneven color and brightness of fundus images, physical model-based methods often have unstable detection effects and low detection accuracy. Classification-based methods tend to have high accuracy because a large number of features are extracted from candidate region images. Deep neural networks have been widely used in the field of computer vision; however, MA detection methods based on deep learning may lead to the existence of over-fitting due to the small amount of data. In addition, deep convolutional neural networks have a large number of parameters and are therefore not easy to use clinically.

In this paper, the classifier-based method was used for MA detection. Previous researchers focused on the target salience of candidates. In addition, we also paid attention to the local structure of the target and background, and proposed the novel local structure feature RGD to overcome the shortcomings of the salience features.

## 3. Materials

We conducted experiments using two publicly available datasets: e-ophtha-MA [14] and ROC [15]. The main specifications of the two datasets are summarized in Table 1.

ROC: The Retinopathy Online Challenge (ROC) contains 50 training images and 50 test images, and all MAs were annotated by four experts. The images have different resolutions, ranging from 768 × 576 to 1394 × 1392 pixels with a 45° field of view (FOV). Since the test images do not have MA annotations, only 50 training images were used to verify our proposed algorithm.

E-ophtha-MA: The e-ophtha-MA is a public dataset of color fundus images designed for scientific research in red lesion (MA and small hemorrhage) detection. It contains 233 healthy images and 148 DR images with four resolutions, ranging from 1440 × 960 to 2544 × 1696 pixels with 45° FOV. All images are used to verify our proposed algorithm.

To assess the ability of the new structural features proposed in this paper, the results of the classification between MAs and non-MAs can be evaluated by the receiver operating characteristic (ROC) curve [16] by plotting the true positive rate (TPR) against the false positive rate (FPR) and the area under the ROC curve (AUC). Different TPR and FPR values can be obtained with different thresholds. They are defined as:

(1) $$TPR=\frac{TP}{P}$$

(2) $$FPR=\frac{FP}{N}$$

where *P* and *N* correspond to the number of MAs and non-MAs in candidates, respectively. TP is the number of MAs correctly detected, FP is the number of MAs incorrectly detected.

In addition, we evaluated the performance of the detection algorithm at the lesion level. The free-response operating characteristic (FROC) curve [17] was used to evaluate the lesion level of MA detection results of all color images. The abscissa of FROC curve is the average number of false positives per image (FPI), and the ordinate is the sensitivity. The sensitivity represents the proportion of MAs correctly detected by the algorithm. These are calculated as follows:

(3) $$Sensitivity=\frac{TP}{N_{MA}}$$

(4) $$FPI=\frac{FP}{N_{i}}$$

where $N_{MA}$ is the number of MAs in all fundus images in the test dataset, $N_{i}$ is the number of images in the test dataset, TP is the number of MAs correctly detected, FP is the number of MAs incorrectly detected. By setting the threshold to classify MA and non-MA, we can obtain pairs <FPI,Sensitivity> to draw the FROC curve.

Under the same FPI, higher sensitivity means better detection performance. Similarly, under the same sensitivity, lower FPI means fewer misdetected MA in one image. In order to compare with different methods, we obtained the sensitivity values from FROC curve as the FPI values are 1/8, 1/4, 1/2, 1, 2, 4, and 8. In addition, the average of sensitivity at these seven predefined FPIs ($F_{score}$) and the partial area under FROC curves between 1/8 and 8 FPI normalized by dividing with the maximum FPI ($F_{AUC}$) were obtained as the comprehensive evaluation indexes.

Under different detection tasks, $F_{score}$ and $F_{AUC}$ often have different optimal ranges. Even under the same detection task, the number of images also has a great influence on them. In the ROC dataset, the optimal value of these two evaluation indexes should be greater than 0.4, while in the e-ophtha-MA dataset, it should be greater than 0.5

## 4. Methods

**Figure 2:**
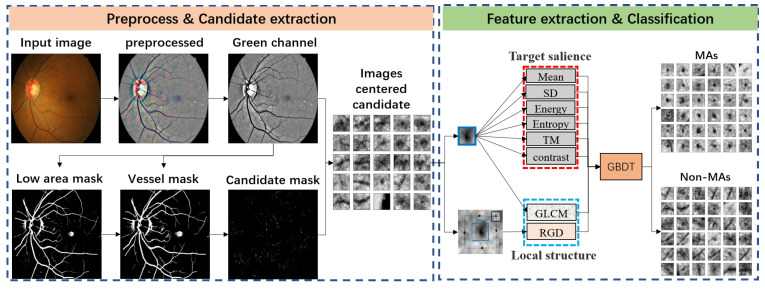
Schematic diagram of the automatic MA detection method, including preprocessing, candidate extraction, feature extraction, and classification.

A schematic diagram of our method is illustrated in Figure 2. It includes three parts: First, the original image was preprocessed to eliminate the interference of uneven color (see Section 4.1). Second, we extracted the MA candidates (see Section 4.2). Finally, a patch centered on each region was collected to extract target salience and local structural features.The target salience include mean, standard deviation (SD), third moment (TM), energy, entropy, and contrast. The local structural features include the texture feature based on Gray level co-occurrence matrix (GLCM) and RGD. Then, we used the combined features to classify candidates to MAs and non-MAs (see Section 4.3).

### 4.1. Image Preprocessing

Due to the interference of environment and equipment in the process of retinal image acquisition and the differences of people’s age and ethnicities [18], the captured fundus images often generally have nonuniform illumination and different colors.

In order to reduce the subsequent computation, we first resized the input image with a scaling factor χ/1400, where χ is related to the width in pixels of the input image. Subsequently, a novel approach proposed by Grinsven et al. [19] was utilized on each channel to enhance the original fundus image with the following equation:

(5) $$I\left(i,j;\sigma \right)=\alpha \cdot I\left(i,j\right)+\tau \cdot Gaussian\left(i.j;\sigma \right)*I\left(i,j\right)+\gamma$$

where ∗ is a convolution operator, σ is the standard deviation of the Gaussian filter, α, τ, and γ are constants. These parameters were set following Grinsven et al. [19], i.e., α=4, τ=−4, γ=128 and σ=χ/30.

**Figure 3:**
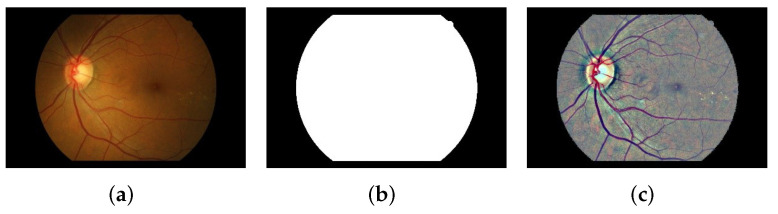
Illustration of the entire procedure for image preprocessing. (**a**) Original resized image. (**b**) Binary mask of the ROI. (**c**) The preprocessed image.

After the image enhancement, there is a great deal of noise in the retina edge. In order to eliminate this noise, we performed region of interest (ROI) detection on it; As shown in Figure 3c, the binary mask of the ROI was obtained by threshold segmentation. Finally, the preprocessed image $I_{ce}$ was measured by means of morphology operations as given in Equation (6):

(6) $$I_{ce}\left(i,j\right)=I_{uni}\left(i,j\right)\cdot \varepsilon^{B}\left(G_{mask}\right)$$

where $I_{uni}$ and $G_{mask}$ correspond to the enhanced image and ROI mask, respectively. $\varepsilon^{B}$ denotes the erosion of an image performed by structural element *B* with disc type. Figure 3 illustrates the entire preprocessing procedure.

### 4.2. Candidate Extraction

**Figure 4:**
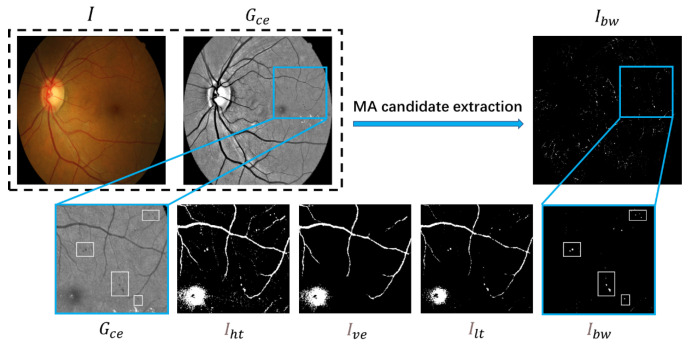
An example to illustrate the candidate extraction method. From top to bottom and left to right: original image *I*, the green channel of the preprocessed image $G_{ce}$, the binary mask of candidate area $I_{bw}$, details in the green channel of the preprocessed image $I_{ce}$, the first binary image $I_{ht}$ under a higher threshold, the vessels mask $I_{ve}$, the second binary image $I_{lt}$ under a lower threshold, and the binary mask of candidate area $I_{bw}$. The areas with MAs are identified with white boxes.

After color correction, the gray value of fundus image is more uniform, which is more conducive to the extraction of the lesion area by threshold segmentation. An effective candidate extraction method should capture MAs as much as possible and capture fewer non-MAs. To accomplish this, we propose a novel candidate extraction algorithm based on dual-gray threshold segmentation and morphological processing. Figure 4 illustrates the entire candidate extraction procedure.

Due to the main information of microaneurysm being in the green channel [3], we first extracted the green channel $G_{ce}$ from $I_{ce}$. As the main non-MAs in the retinal image come from blood vessels, we first performed vessel segmentation.

We obtained the first binary image $I_{ht}$ of the low-gray area through threshold segmentation with a higher gray threshold $T_{h}$ with vessels with more connectivity. The value of $T_{h}$ ranges from 100 to 115. Then, we reserve the connected domain with an area greater than *S* through connected domain analysis. The retinal blood vessels mask $I_{ve}$ can be obtained by Equation (7):

(7) $$I_{ve}\left(i,j\right)=\left\{\begin{array}{cc} I_{ht}\left(x,y\right) & \;\mathrm{if}\;s(x,y)>S\\ 0 & \;\mathrm{if}\;s(x,y)\leq S \end{array}\right.$$

where $I_{ve}$ corresponds to the vessel mask, and $I_{ht}$ corresponds to the first binary image. s(x,y) correspond to the area of the connected domain in which pixel (x,y) is located. As the retinal vessels occupy a large area, the value of *S* is set to 400.

After the vessel mask $I_{ve}$ is obtained, we expanded $I_{ve}$ to ensure that the vessel edge can also be eliminated. In addition, we obtained the second binary image $I_{lt}$ with a lower threshold $T_{l}$ through threshold segmentation, so that fewer non-MAs can be captured. The value of $T_{l}$ ranged from 90 to 100. Then, the binary mask of candidate area $I_{bw}$ was obtained according to the following equation:

(8) $$I_{bw}=I_{lt}\cdot \left(1-\varepsilon^{B}\left(I_{ve}\right)\right)$$

where $I_{bw}$ and $I_{lt}$ correspond to the binary mask of candidate area and the vessel mask, respectively. $\varepsilon^{B}\left(\right)$ denotes erosion of an image performed by rectangular element *B* with size k×k. In this article, k=5. Then, we conducted the connected domain analysis for $I_{bw}$ and deleted the pixels whose connected domain area was equal to 1.

We considered each of the connected domains in the binary image $I_{BW}$ as a candidate region for the possible existence of MA. Consequently, we use the center coordinates of each connected domain to extract a certain size image from the preprocessed image $I_{ce}$ for feature extraction and target recognition.

### 4.3. Feature Extraction and Classification

The salience and local structural features were extracted from each candidate. The local structure features include local texture based on Gray level co-occurrence matrix (GLCM) and our proposed RGD. Then, we combined all the features and used GBDT to classify MA candidates.

#### 4.3.1. Object Salience

Saliency features of objects have been widely used in classification and detection tasks [20,21,22,23,24]. Since different candidate regions have different sizes and shapes, we extracted image patches of a certain size, which is sufficient to contain one MA from the center of each candidate region as the source of salience features. The size is set to 11×11. In this study, six salience features were extracted from the green channel, including the mean, standard deviation(SD), third moment (TM), energy, entropy, and contrast [25,26,27].

#### 4.3.2. Local Structures

Local structures include the local texture and RGD. The local texture indicate the homogeneity information of objects [28,29,30,31], which is calculated depending on the pixels and their surroundings [32]. We implemented texture feature extraction based on GLCM [33] and six features based on Haralick features [34] were obtained on one offset GLCM matrix. In our work, we selected four different offsets (0${}^{\circ }$, 45${}^{\circ }$, 90${}^{\circ }$, 135${}^{\circ }$) resulting in 24-dimensional Haralick features.

The local texture features used in this article are shown in Table 2. p(i,j) correspond the (i,j)th entry in normalized GLCM, $p_{x}\left(i\right)$ correspond *i*th entry in the marginal-probability matrix obtained by summing the rows of p(i,j), $p_{y}\left(j\right)$ correspond *j*th entry in the marginal-probability matrix obtained by summing the columns of p(i,j), and $\mu_{x}$, $\mu_{y}$, $\sigma_{x}$, and $\sigma_{y}$ are the means and standard deviations of $p_{x}$ and $p_{y}$. $p_{x-y}=\sum_{i=1}^{N_{g}}\sum_{j=1}^{N_{g}}p\left(i,j\right)$, $\left|i-j\right|=0,1,\ldots ,N_{g}-1$, and $N_{g}$ is the number of gray levels.

The object salience and local texture ignore the relationship between the target and the surrounding background, so their description ability is not enough. By introducing the surrounding background, we can observe a large degree of structural difference between MA and non-MA in this local region.

**Figure 5:**
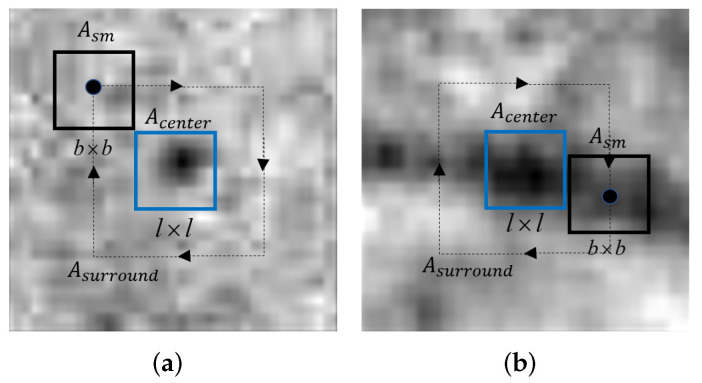
Examplesof candidate images. (**a**) Candidate image containing an MA. (**b**) Candidate image containing a vessel.

Vessels are the major component of non-MAs. We defined the l×l area in the center of the candidate area as the central area $A_{ce}$, and the adjacent area with width *b* as the surrounding area $A_{sr}$. As shown in Figure 5, if the target contained in the candidate region image is MA, we can see that the surrounding region is the retinal background. If the target of the candidate region image is a blood vessel, we can always find a region with the lowest gray mean similar to the central region in the surrounding region. Therefore, we propose a novel local structure feature called a ring gradient descriptor (RGD) to calculate the minimum gradient between the candidate image block and its surroundings to distinguish MAs from vessels.

First, we find the region with the minimum gray mean $A_{sm}$ in the surrounding region in the area around the target through annular scanning, and the size of the scan box is $A_{sm}$ is b×b. Since the blood vessels are multi-directional, we set the moving step of the scan box to 1 pixel each time. Then, the number of scanning *K* can be calculated as 4(b+l) and the minimum gradient between $A_{ce}$ and $A_{sm}$ can be calculated by the following equation:

(9) $$RGD=\frac{1}{b^{2}}\sum_{i=1}^{b}\sum_{j=1}^{b}A_{sm}\left(i,j\right)-A_{ce}\left(i,j\right)*G\left(i,j;\sigma \right)$$

where G(i,j;σ) is the Gaussian kernel with standard deviation σ, which has the same size as the central region. ∗ is the convolution operator. Since the gray level of MA is lower in the center and higher in the surrounding area, Gaussian convolution is used instead of calculating the gray level mean, mainly to control the weight of each pixel.

**Figure 6:**
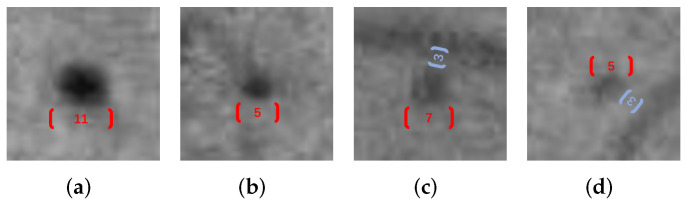
Illustrative example of the optimal parameter settings of different candidates. (**a**–**d**) are MAs in different environments, with different pixel widths and distances from the adjacent blood vessel. These two values are marked by red and blue markers respectively.

The two most important parameters in the RGD algorithm are *l* and *b*, which are determined by the size of MA and the distance from the surrounding blood vessels. The value of σ transforms with *l*. As shown in Figure 6, different candidates have different optimal parameters. In order to make the model more robust, we calculated 10 RGDs under different parameters as new local structural features as shown in Table 3.

#### 4.3.3. Classify

To distinguish between MAs and non-MAs, the GBDT classifier [35], which is an ensemble classifier that has been used in general applications, was employed in our work. The increasing popularity of this classifier is mainly attributed to its faster training speed and its robustness. As introduced before, a training set $S_{GB}=\left\{x^{\left(i\right)},y^{\left(i\right)}\right\},i=1,2,\ldots ,N$ is constructed by combining features *x* and corresponding label.

**Figure 7:**
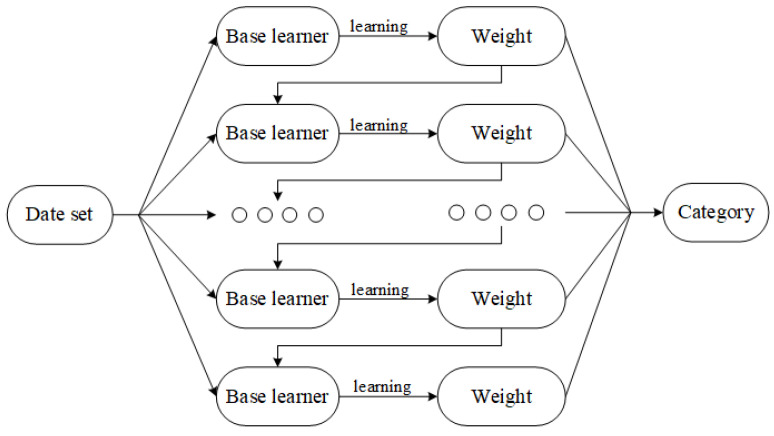
The construction of GBDT [36].

The establishment process of the GBDT is shown in Figure 7, It consists of *M* base classifiers. The base learner of GBDT is the classification and regression tree (CART). The complete algorithm process of GBDT binary classification algorithm is as follows:

(1) Initialize the first weak CART:

(10) $$F_{0}\left(x\right)=log\frac{P(Y=1|x)}{1-P(Y=1|x)}$$

where P(Y=1|x) is the proportion of MAs in the training sample.

(2) Perform m(m=1,2,3…M) iterations on the base learner:

For i=1,2,…,N, calculate the response value corresponding to the *m*th tree (negative gradient of the loss function):

(11) $$r_{m,i}=y_{i}-\frac{1}{1+e^{-F_{m-1}\left(x_{i}\right)}}$$

For i=1,2,…,N, use CART regression tree fitting data $\left(x_{i},r_{m,i}\right)$ to get the *m*th regression tree, whose corresponding leaf node area is $R_{m,j}$, where $j=1,2,\ldots ,J_{m}$ and $J_{m}$ is the number of leaf nodes of the *m*th regression tree.

For $J_{m}$ leaf node region $j=1,2\ldots ,J_{m}$, the best fitting value $c_{m,j}$ was calculated:

(12) $$c_{m,j}=\frac{\sum_{x_{i}\in R_{m,j}}r_{m,i}^{}}{\sum_{x_{i}\in R_{m.j}}\left(y_{i}-r_{m,i}\right){\left(1-y_{i}+r_{m,i}\right)}^{}}$$

Update the strong classifier $F_{m}\left(x\right)$:

(13) $$F_{m}\left(x\right)=F_{m-1}\left(x\right)+\sum_{j=1}^{J_{m}}c_{m,j}I\left(x\in R_{m,j}\right)$$

(3) Then, the final strong classifier $F_{M}\left(x\right)$ can be expressed as:

(14) $$F_{M}\left(x\right)=F_{0}\left(x\right)+\sum_{m=1}^{J_{m}}\sum_{j=1}^{J_{m}}c_{m,j}I\left(x\in R_{m,j}\right)$$

(4) Finally, the classification value of sample *x* can be expressed as:

(15) $$P\left(Y=1|x\right)=\frac{1}{1+e^{-F_{M}\left(x\right)}}$$

The learning rate of model (ε) and the number of levels of trees (*C*) are the hyperparameters that have the most obvious impact on the accuracy of GBDT model. The prediction accuracy of GBDT is significantly affected by the value of hyperparameter [36]. After plenty of experimentation, the optimal values of ε and *C* were set to 0.03 and 80, respectively.

## 5. Results

### 5.1. Candidate Extraction Evaluation

The performance of candidate extraction proposed in this article was evaluated by sensitivity and compared with the previously published candidate extractor algorithms as demonstrated in Table 4. The proposed method achieved a sensitivity value of 0.51 for the ROC dataset. Although this value did not reach the maximum value, it had a relatively low FPI value, and the performance of the candidate extraction algorithm proposed is better than some algorithms. We obtained a sensitivity value of 0.72 in the ephtha-MA dataset, corresponding to an FPI value of 200.74.

### 5.2. Candidate Classification Evaluation

**Figure 8:**
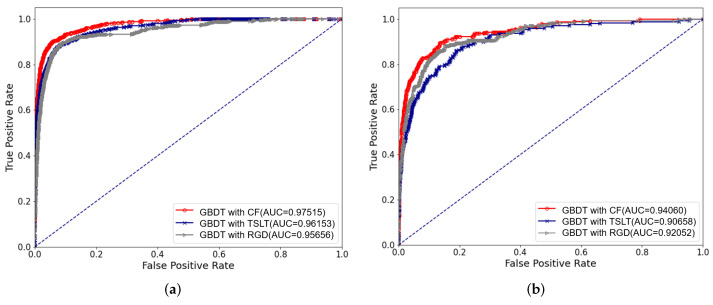
ROC curves of MAs and non-MAs classification on different features. (**a**) The results of the e-optha-MA dataset. (**b**) The results of the ROC dataset.

We adopted five-fold cross-validation for model training. We trained three models using target saliency and local texture (TSLT), RGD, and the combined features (CF). The performance of classification based on object salience features and local structure features was evaluated by ROC curve as shown in Figure 8. Their AUC values are shown in Table 5.

The combined features achieved much higher AUC (AUC = 0.9752 in e-optha-MA, AUC = 0.9409 in ROC) than using traditional TSLT features individually (AUC = 0.9615 in e-optha-MA, AUC = 0.9066 in ROC). The AUC value (AUC = 0.9566) obtained using RGD features were close to those obtained using TSLT features (AUC = 0.9615) in dataset e-optha-MA, and the AUC values (AUC = 0.9205) obtained by using RGD features in dataset ROC exceeded those (AUC = 0.9066) obtained by using TSLT features alone. Therefore, combining traditional features with RGD can improve the classification performance of the model.

**Figure 9:**
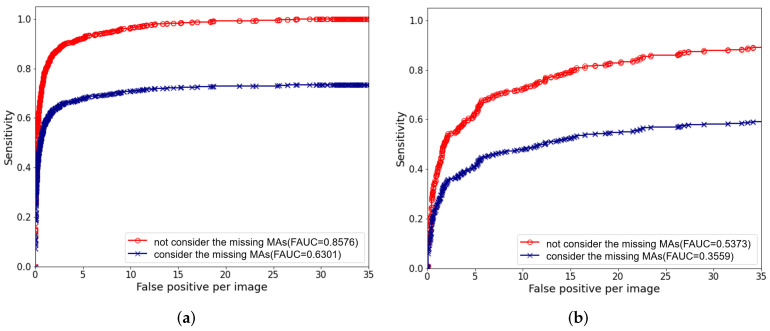
FROC curves of MAs and non-MAs classification. (**a**) The results of the e-optha-MA dataset. (**b**) The results of the ROC dataset.

Figure 9 presents the FROC curves obtained by not considering the missing MAs from the candidate extraction step and consider. Since many MAs were omitted in the extraction algorithm of candidates, the $F_{socre}$ and $F_{AUC}$ values of the final model would be reduced. The values of $F_{score}$ and $F_{AUC}$ in dataset e-optha-MA without considering the omission of MA in the second step were 0.591 and 0.794, which are much higher than the values of 0.434 and 0.583 considering the omission of MA, and the values of 0.349 and 0.519 in dataset ROC are higher than the values of 0.188 and 0.280.

The final results after candidate extraction and candidate classification at the lesion level were compared with other MA detection methods in the ROC and e-ophtha MA datasets as shown in Table 6. After verification, our algorithm is ahead of some other algorithms in the ROC dataset ($F_{score}=0.264$, $F_{AUC}=0.356$), and the $F_{AUC}$ and $F_{score}$ values achieved a leading level in the e-ophtha MA dataset ($F_{score}=0.547$, $F_{AUC}=0.630$). Although we achieved a high detection performance in the e-ophtha MA datasets, the result in the ROC dataset was poor because of the simplicity of the candidate extraction algorithm.

The sensitivity values of 0.51 and 0.72 in the ROC and e-ophtha MA datasets were achieved in the candidate extraction step, respectively, which can be said to be the upper limit of the final detection algorithm. The proposed method achieved the sensitivity values of 0.468 and 0.696, respectively, at the two datasets where FPIs is 8, which were close to the upper limit. This shows that our classification algorithm has high performance and the features we extracted were very effective.

**Figure 10:**
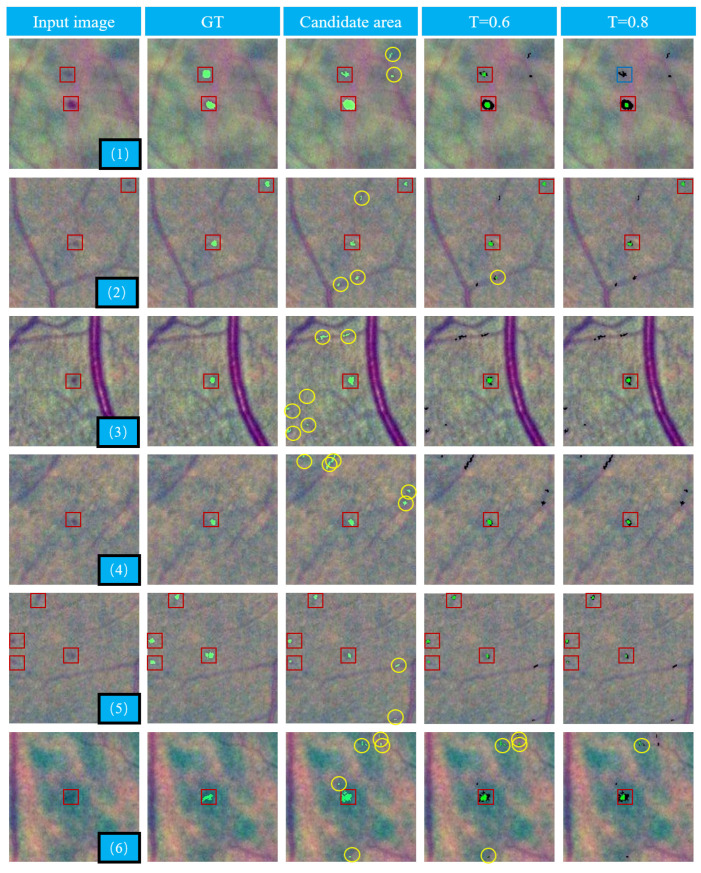
Illustration of pixel level validation. For better visualization, the correctly detected MAs and miss detection MAs are highlighted by red and blue squares, respectively. The FP candidates are highlighted by yellow circles.

In order to more qualitatively display the performance of RGD on MA detection, we used six 224 × 224 color images containing MAs for pixel-level validation, as shown in Figure 10. The MA candidate region was obtained through dual-threshold segmentation proposed by us. RGD was performed for each pixel in the MA candidate region and the values of *b*, *l*, and σ are 5.7, and 0.6, respectively. Then, we normalized the results and segmented them with thresholds of 0.6 and 0.8, respectively. We found that the use of RGD alone was also effective to detect MAs.

## 6. Discussion

In this paper, we proposed an efficient local structure awareness-based retinal MA detection method with the multi-feature combination (LSAMM). First, the color correction was performed on the images, and simple morphology and threshold segmentation method was used to extract MA candidates. The sensitivity values of this stage in the ROC and e-ophtha MA dataset, respectively, were 0.51 and 0.72, and their corresponding FPI values were 243.38 and 200.74, respectively.

In the candidate classification step, a novel local structure feature RGD was proposed that can effectively distinguish MA and vessels and improve the performance of classification. After using this, the AUC value in the e-ophtha MA dataset increased from 0.96153 to 0.97515, and in the ROC dataset, the AUC value increased from 0.90658 to 0.94060. The whole MA detection algorithm proposed achieved a high detection performance in the e-ophtha MA dataset ($F_{score}=0.567$); however, its performance in the ROC dataset was mediocre ($F_{score}=0.264$) as the sensitivity value in candidate extraction step was low.

The candidate extraction algorithm performed worse in the ROC dataset than in the e-ophtha MA dataset. Nevertheless, the results of candidate classification showed high performance in both datasets. The detection performance of MA can be improved by changing the candidate extraction algorithm and combining RGD with other conventional features in the candidate classification step.

## Figures and Tables

**Table 1:** Dataset specifications.

	Image Size (px)	FOV (Degree)	FOV Diameter (px)	NE	NIN	NIP	NMA
ROC	768 × 576, 1394 × 1392	45°	720–1345	4	13	37	336
e-ophtha-MA	1440 × 960, 2544 × 1966	45°	910–1925	1	233	148	1306

FOV: field of view. NE: number of experts. NIN: number of images without MAs. NIP: number of images with MAs. NMA: total number of MAs.

**Table 2:** The local texture-feature-based GLCM matrix [34].

Feature Name	Description
correlation	$\sum_{i}\sum_{j}\frac{\left(ij\right)p\left(i,j\right)-\mu_{x}\mu_{y}}{\sigma_{x}\sigma_{y}}$
inverse difference moment	$\sum_{i}^{}\sum_{j}^{}\frac{1}{1+{\left(i-j\right)}^{2}}p\left(i,j\right)$
difference variance	variance of $p_{x-y}$
entropy	$-\sum_{i}^{}\sum_{j}^{}p\left(i,j\right)log\left(p\left(i,j\right)\right)$
angular moment	$\sum_{i}\sum_{j}{\left(p\left(i,j\right)\right)}^{2}$
contrast	$\sum_{i}^{}\sum_{j}^{}{\left(i-j\right)}^{2}p\left(i,j\right)$

**Table 3:** Local structural features.

Parameters	Description										
b	The width of the surrounding area	3	3	3	3	3	5	5	5	5	5
l	The width of the central area	5	7	9	11	13	5	7	9	11	13
σ	The standard deviation of Gaussian kernel	0.9	0.7	0.5	0.5	0.5	0.9	0.7	0.5	0.5	0.5

**Table 4:** Candidate extraction performance using the ROC dataset.

Method	Sensitivity	FPI
Proposed method	0.51	243.38
Shah et al. [7]	0.48	65.00
Dai et al. [37]	0.69	569.39
Adal et al. [38]	0.45	35.2
Walter et al. [39]	0.36	154.42
Zhang et al. [40]	0.33	328.30
Dashtbozorg et al. [4]	0.82	755.50

**Table 5:** Validation of different features in MA candidate classification.

Database	Method	AUC
e-optha-MA	TSLT	0.9615
	RGD	0.9566
	CF	0.9752
ROC	TSLT	0.9066
	RGD	0.9205
	CF	0.9409

**Table 6:** Performance of different MA detection methods in the ROC and e-ophtha MA datasets.

Database	Work	Sensitivty against FPIs							$F_{\mathrm{score}}$	$F_{\mathrm{AUC}}$
ROC		1/8	1/4	1/2	1	2	4	8		
	Proposed work	0.083	0.104	0.200	0.257	0.344	0.394	0.468	0.264	0.356
	Chudzik et al. [41]	0.039	0.067	0.141	0.147	0.243	0.306	0.385	0.193	-
	Dashtbozorg et al. [4]	0.435	0.443	0.454	0.479	0.481	0.495	0.506	0.471	0.484
	Eftekhari et al. [42]	0.047	0.173	0.351	0.552	0.613	0.722	0.769	0.461	0.660
	Wu et al. [43]	0.037	0.056	0.103	0.206	0.295	0.339	0.376	0.202	-
	Budak et al. [13]	0.039	0.061	0.121	0.220	0.338	0.372	0.394	0.221	-
	Wang et al. [44]	0.273	0.379	0.398	0.481	0.545	0.576	0.598	0.464	-
	Dai et al. [37]	0.219	0.257	0.338	0.429	0.528	0.598	0.662	0.433	0.553
	Antal and Hjdu [6]	0.173	0.275	0.380	0.444	0.526	0.599	0.643	0.434	0.551
	Melo et al. [5]	0.053	0.066	0.077	0.098	0.146	0.208	0.259	0.130	0.185
e-ophtha-MA	Proposed work	0.335	0.424	0.496	0.578	0.634	0.668	0.696	0.547	0.630
	Wu et al. [43]	0.063	0.117	0.172	0.245	0.323	0.417	0.573	0.273	-
	Dashtbozorg et al. [4]	0.358	0.417	0.417	0.522	0.558	0.605	0.638	0.510	0.575
	Eftehari et al. [42]	0.091	0.258	0.401	0.534	0.579	0.667	0.771	0.471	0.637
	Chudzik et al. [41]	0.185	0.313	0.465	0.604	0.716	0.801	0.849	0.562	-
	Melo et al. [5]	0.178	0.284	0.383	0.519	0.587	0.587	0.587	0.446	0.551

## Data Availability

All data are available from the corresponding author upon request.
